# P-2354. Neonatal Enterovirus and Parechovirus Infections—National Enterovirus Surveillance System, United States, 2004–2022

**DOI:** 10.1093/ofid/ofae631.2505

**Published:** 2025-01-29

**Authors:** Miranda Delahoy, Halle Getachew, Randall English, Sarah Kidd

**Affiliations:** Centers for Disease Control and Prevention (CDC), Atlanta, Georgia; Centers for Disease Control and Prevention, Atlanta, Georgia; Tanaq Management Services LLC, Atlanta, Georgia; Centers for Disease Control and Prevention, Atlanta, Georgia

## Abstract

**Background:**

Enteroviruses (EV)—a group of viruses including echoviruses (E) and coxsackieviruses (CV)—and parechoviruses (PeV) can cause a range of symptoms, including respiratory illness; hand, foot, and mouth disease; acute flaccid myelitis; meningitis; and sepsis. Neonates (persons < 1 month old) are at higher risk of severe EV or PeV disease. We analyzed data from the National Enterovirus Surveillance System (NESS) to assess types and outcomes of neonatal EV and PeV infections reported during 2004–2022.

**Methods:**

NESS is a passive, laboratory-based surveillance system that collects reports of positive EV and PeV virus type results in the United States. We analyzed data on EV and PeV specimens collected during January 1, 2004–December 31, 2022. Outcome data (defined as whether the patient died) were reported to NESS during 2014–2022. Chi-squared tests were used to compare virus type frequencies by age, with differences considered significant if *p* < 0.05.

**Results:**

Overall, 10,833 EV and PeV infections were reported during 2004–2022: 9,197 (85%) had reported age, of which 753 (8%) were among neonates. Among 631 neonatal EV or PeV infections with identified virus type (84%), Coxsackievirus type B5 (CVB5) (76; 12%), CVB3 (64; 10%), PeVA3 (61; 10%), E11 (54; 9%), and CVB4 (52; 8%) were detected most frequently. Compared with persons ≥ 1 month old, CVB types 1–5, PeVA3, and E11 were significantly more likely to be detected among neonates, whereas EV-D68 and E30 infections were less common. Among 483 neonates with EV or PeV infections during 2014–2022, 68 (14%) had known outcome, of whom 16 (24%) died.

**Conclusion:**

EV and PeV infections can cause severe disease among neonates. The frequencies of EV and PeV virus types detected among neonates differ from those among persons ≥1 month old, although this may partially reflect testing differences by age. Assessing mortality among neonates with EV and PeV illness is limited by incomplete outcome data and lack of systematic EV and PeV testing and reporting, which may be biased toward including patients with more severe illness. Strengthening capacity for EV and PeV testing, typing, and surveillance would be beneficial for understanding the disease burden among neonates and informing treatment options and prevention measures.

**Disclosures:**

All Authors: No reported disclosures

